# Assigning strains to bacterial species via the internet

**DOI:** 10.1186/1741-7007-7-3

**Published:** 2009-01-26

**Authors:** Cynthia J Bishop, David M Aanensen, Gregory E Jordan, Mogens Kilian, William P Hanage, Brian G Spratt

**Affiliations:** 1 Department of Infectious Disease Epidemiology, Imperial College London, St. Mary's Hospital Campus, London, UK; 2 European Bioinformatics Institute, Wellcome Trust Genome Campus, Hinxton, Cambridge, UK; 3 Department of Medical Microbiology and Immunology, Aarhus University, DK-8000 Aarhus, Denmark

## Abstract

**Background:**

Methods for assigning strains to bacterial species are cumbersome and no longer fit for purpose. The concatenated sequences of multiple house-keeping genes have been shown to be able to define and circumscribe bacterial species as sequence clusters. The advantage of this approach (multilocus sequence analysis; MLSA) is that, for any group of related species, a strain database can be produced and combined with software that allows query strains to be assigned to species via the internet. As an exemplar of this approach, we have studied a group of species, the viridans streptococci, which are very difficult to assign to species using standard taxonomic procedures, and have developed a website that allows species assignment via the internet.

**Results:**

Seven house-keeping gene sequences were obtained from 420 streptococcal strains to produce a viridans group database. The reference tree produced using the concatenated sequences identified sequence clusters which, by examining the position on the tree of the type strain of each viridans group species, could be equated with species clusters. MLSA also identified clusters that may correspond to new species, and previously described species whose status needs to be re-examined. A generic website and software for electronic taxonomy was developed. This site  allows the sequences of the seven gene fragments of a query strain to be entered and for the species assignment to be returned, according to its position within an assigned species cluster on the reference tree.

**Conclusion:**

The MLSA approach resulted in the identification of well-resolved species clusters within this taxonomically challenging group and, using the software we have developed, allows unknown strains to be assigned to viridans species via the internet. Submission of new strains will provide a growing resource for the taxonomy of viridans group streptococci, allowing the recognition of potential new species and taxonomic anomalies. More generally, as the software at the MLSA website is generic, MLSA schemes and strain databases for other groups of related species can be hosted at this website, providing a portal for microbial electronic taxonomy.

## Background

The methods used to assign strains to bacterial species, and the definition of species, have been much debated [1-4]. There is widespread recognition that the current methods of defining prokaryotic species are no longer adequate and there has been little progress in developing an agreed concept of species that can guide a new definition of species [5-7]. The current approach for distinguishing species within a genus, and for defining new species, is based on polyphasic taxonomy, which incorporates all available phenotypic and genotypic data into a consensus classification [8]. The accepted genotypic method for defining species is based on overall genomic relatedness, such that strains which share approximately 70% or more relatedness using DNA-DNA hybridization, with 5°C or less Δ*T*_m _(the difference in the melting temperature between homologous and heterologous hybrids), under standard conditions, are considered to be members of the same species [9]. The cut-off value implied by this definition may not be appropriate for all bacterial populations and in recent years a move away from this cumbersome procedure to other ways of identifying and circumscribing bacterial species has been encouraged [10].

One approach is to observe the distribution of a large number of strains of closely related species in sequence space and to identify clusters of strains that are well resolved from other clusters. This approach has been developed by using the concatenated sequences of multiple core (house-keeping) genes to assess clustering patterns, and has been called multilocus sequence analysis (MLSA) [2,11] or multilocus sequence phylogenetic analysis [12]. MLSA has been used successfully to explore clustering patterns among large numbers of strains assigned to very closely related species by current taxonomic methods [12-18], to look at the relationships between small numbers of strains within a genus [19], or within a broader taxonomic grouping [20], and to address specific taxonomic questions [21,22]. More generally, the method can be used to ask whether bacterial species exist – that is, to observe whether large populations of similar strains invariably fall into well-resolved clusters, or whether in some cases there is a genetic continuum in which clear separation into clusters is not observed.

Although there are limitations of the MLSA approach [11], it is attractive since it should allow the assignment of unknown strains to species clusters via the internet (electronic taxonomy), in a similar way that isolates have been assigned to strains (sequence types) or clonal complexes within a species using multilocus sequence typing (MLST) [23]. Electronic taxonomy requires the recognition and circumscription of species by the generation of a large initial database (and associated MLSA data) of multiple strains from all of the recognized species within a genus, or part of a genus. It also requires a website and software that allow the sequences from the house-keeping genes of a new query strain to be entered via the internet, and for the sequence cluster (if any) within which the query strain falls to be returned, along with a species assignment for the strain. The approach therefore allows species assignment via the internet and also allows the recognition of the appearance in the database of new clusters, which can be assigned as new candidate species.

We describe an exemplar for electronic taxonomy (and a generic website) using the viridans group streptococci, since the taxonomy of this group has been, and to some extent remains, problematic [24]. The non-pyogenic streptococci are subdivided into the Mitis, Anginosus, Salivarius, Mutans and Bovis groups, of which the first three are often referred to as viridans streptococci. The Mitis group currently includes the important pathogen *S. pneumoniae *and 12 other validly described species, *S. australis, S. cristatus *(formerly *S. crista*), *S. gordonii*, *S. infantis, S. mitis*, *S. oligofermentans*, *S. oralis*, *S. parasanguinis *(formerly *S. parasanguis*), *S. peroris*, *S. pseudopneumoniae*, *S. sanguinis *(formerly *S. sanguis*) and *S. sinensis*. The Anginosus group includes three recognized species, *S. anginosus*, *S. constellatus *(including two subspecies *subsp. constellatus *and *subsp. pharyngis*) and *S. intermedius*, and the Salivarius group includes *S. salivarius*, *S. vestibularis*, and *S. thermophilus*. All viridans streptococci (with the exception of *S. thermophilus*, which is not associated with humans) are commensals of the human upper respiratory tract and, excepting *S. pneumoniae*, rarely cause disease in immune-competent individuals. They have been strikingly resistant to satisfactory classification, reflected in frequently changing nomenclature and significant problems of identification by phenotypic analysis and by sequencing of 16S rRNA genes [16,25-33].

There is extensive evidence that recombination occurs between closely related Mitis group species [34-38], which can lead to fuzzy species [14], and may explain some of the taxonomic confusion within this group, and could prevent the identification of well-resolved sequence clusters. However, initial studies of the Mitis group using MLSA have shown that sequence clusters corresponding to *S. pneumoniae*, *S. pseudopneumoniae*, *S. mitis *and *S. oralis *can be resolved using trees constructed from the concatenated sequences of six of the seven housekeeping genes used in the *S. pneumoniae *MLST scheme [11,39]. Similarly, concatenation of the sequences of four house-keeping genes has been shown to resolve the more distantly related species within the viridans streptococci [16], although with four genes there was poor resolution between the strains of the closely related species, *S. pseudopneumoniae *and *S. mitis *[18].

In this study, the sequences of a set of seven house-keeping gene fragments were obtained from 420 strains, including strains of all species within the Mitis, Anginosus and Salivarius group streptococci, to develop a viridans group MLSA scheme and strain database. A generic open-access MLSA website  was developed that allows query strains to be assigned via the internet to known species clusters, to as yet unnamed sequence clusters, or as single divergent genotypes.

## Results

### Amplification and sequencing of gene fragments from viridans group streptococci

The primers for the amplification of internal fragments of eight house-keeping gene fragments were selected as in Methods (Table 1). The primers successfully amplified the eight gene fragments from 326 of the 375 (87%) Mitis group strains that were examined. They also amplified the correct fragments from some streptococcal species outside the Mitis group, including all strains of *S. anginosus*, *S. vestibularis *and *S. pyogenes *that were examined. However, the *guaA *gene could not be amplified from some strains (mostly *S. sanguinis *and *S. cristatus*) and this gene was eliminated to produce the final seven-locus MLSA scheme. The seven selected house-keeping genes could be amplified from all viridans group streptococci that were examined and were at least 40 kb apart on the *S. pneumoniae *R6 chromosome.

**Table 1:** Genes and PCR primers used in viridans group MLSA scheme

Primer	Gene product	Sequence (5'-3')*	Primer length (bp)	Trimmed fragment size (bp)	Annealing temperature (°C)
*guaA*-up	GMP synthase	ATYCARTTYCACCCMGAAGT	20	567	55
*guaA*-dn		CWGGNCCWGGRAATGGTTG	19		55
*map*-up	Methionine aminopeptidase	GCWGACTCWTGTTGGGCWTATGC	23	348	55
*map*-dn		TTARTAAGTTCYTTCTTCDCCTTG	24		55
*pfl*-up	Pyruvate formate lyase	AACGTTGCTTACTCTAAACAAACTGG	26	351	55
*pfl*-dn		ACTTCRTGGAAGACACGTTGWGTC	24		55
*ppaC*-up	Inorganic pyrophosphatase	GACCAYAATGAATTYCARCAATC	23	552	50
*ppaC*-dn		TGAGGNACMACTTGTTTSTTACG	23		50
*pyk*-up	Pyruvate kinase	GCGGTWGAAWTCCGTGGTG	19	492	50
*pyk*-dn		GCAAGWGCTGGGAAAGGAAT	20		50
*rpoB*-up	RNA polymerase beta subunit	AARYTIGGMCCTGAAGAAAT	20	516	50
*rpoB*-dn		TGIARTTTRTCATCAACCATGTG	23		50
*sodA*-up	Superoxide dismutase	TRCAYCATGAYAARCACCAT	20	378	50
*sodA*-dn		ARRTARTAMGCRTGYTCCCARACRTC	26		50
*tuf*-up	Elongation factor Tu	GTTGAAATGGAAATCCGTGACC	22	426	55
*tuf*-dn		GTTGAAGAATGGAGTGTGACG	21		55

*Sequences are shown using the IUPAC codes for sites where degeneracy was introduced.

For each strain, the gene fragments were sequenced on both strands and trimmed to defined start and end positions. For each locus, the trimmed sequences from all strains were the same length, indicating that indels in these genes were absent, as they were in the corresponding *S. agalactiae, S. mutans *and *S. suis *sequences, and are thus likely to be uncommon among the viridans group.

### Identification of sequence clusters

**Figure 1:**
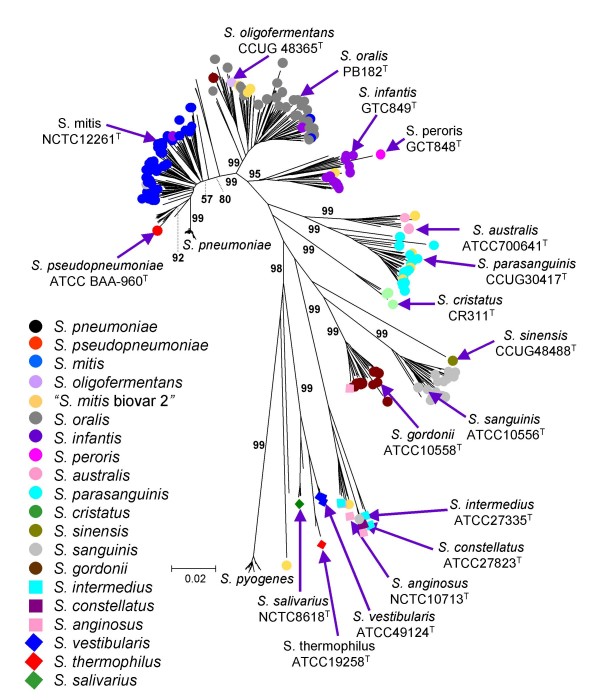
**Tree showing the positions of well-characterized strains and type strains within sequence clusters**. A neighbour-joining radial tree was constructed using the concatenated sequences of all 420 strains. The viridans group strains that are coloured were those assigned to species in the laboratory of MK; the positions on the tree of the type strains of viridans group species are indicated. Mitis, Anginosus and Salivarius group species are shown, respectively, as coloured circles, squares and diamonds. Bootstrap support values (%) for each of the nodes leading to the Mitis group sequence clusters are indicated; values for the clusters within the Anginosus and Salivarius groups are shown in Figure 2C. The colour key is ordered top to bottom according to the position of the clusters on the radial tree, from *S. pneumoniae *to *S. pyogenes*.

Figure 1 shows a neighbour-joining tree for all 420 concatenated sequences (402 from viridans group strains, 17 from strains of *S. pyogenes *and one from *S. agalactiae*) of the seven loci used in the new seven-locus MLSA scheme, with the strains coloured where a clear species assignment was provided by the laboratory of MK. The tree identified a number of sequence clusters that were well resolved, with ≥ 95% bootstrap confidence values for the nodes. The strains identified as *S. pseudopneumoniae *(including the type strain) clustered together but were not well resolved from the *S. mitis *cluster (bootstrap value of 92% for the node separating the *S. pseudopneumoniae *and *S. mitis *clusters on the neighbour-joining tree, but only 46% on a minimum evolution tree; see below). The *S. mitis *cluster was unusual as it consisted of a set of closely related sub-clusters arising from the branch that separates the *S. pneumoniae *and *S. pseudopneumoniae *clusters from that of *S. oralis*.

Concordance between these clusters and the assigned species names was imperfect, in accord with the difficulties in assigning viridans group strains to species. However, almost all of the clusters contained only one designated type strain (Figure 1). Accordingly, the majority of the sequence clusters may be equated with recognized species, that is, *S. anginosus, S. australis, S. constellatus, S. cristatus, S. gordonii, S. infantis, S. intermedius, S. mitis, S. oralis, S. parasanguinis, S. pseudopneumoniae, S. sanguinis*, *S. salivarius, S. thermophilus *and *S. vestibularis*. The only exceptions were the *S. peroris *type strain which, although on an unusually long branch, fell within a cluster that otherwise included the *S. infantis *type strain and several reference strains of that species, and *S. oligofermentans *that was within a subgroup of *S. oralis *(see below).

For several of the sequence clusters there was complete, or almost complete, concordance with species names assigned on the basis of phenotypic examination. However, for some species (for example, *S. mitis, S. infantis *and *S. oralis*) that have been affected by changing taxonomy, or difficulties in phenotypic differentiation, there was significant discordance between the clusters and previously assigned species names. The major anomaly was that strains historically assigned as *S. mitis *fell into a number of different closely related sequence clusters, particularly those otherwise consisting of *S. infantis *and *S. oralis*. This anomaly was due largely to strains initially assigned as '*S. mitis *biovar 2' being either part of the *S. oralis *cluster (see below), in agreement with a recent observation [18], or part of the subsequently described *S. parasanguinis *cluster [26]. Besides the anomalous clustering of some '*S. mitis' *strains, there were single strains assigned as *S. gordonii *and *S. infantis *within the *S. oralis *cluster, a single strain of '*S. mitis *biovar 2' within the *S. anginosus *cluster and a single strain of *S. anginosus *within the *S. constellatus *cluster.

**Figure 2:**
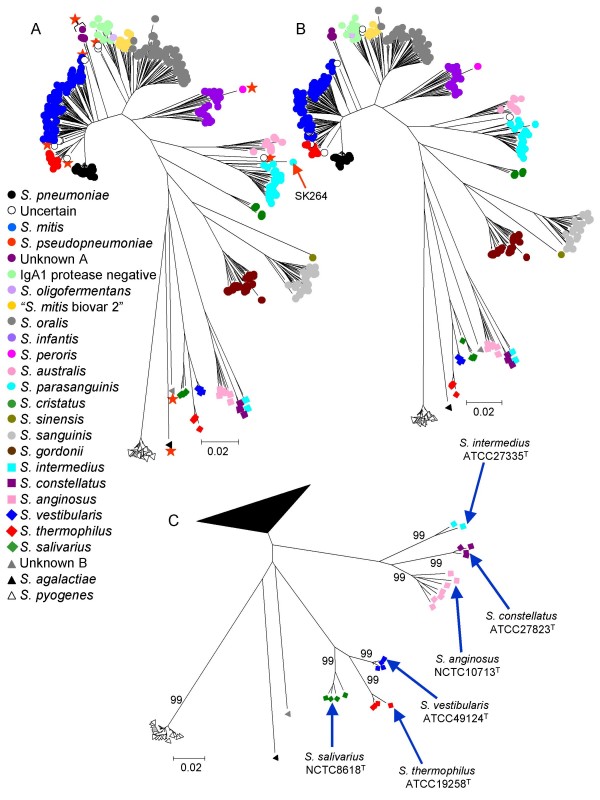
**The MLSA reference tree**. A. The neighbour-joining tree of Figure 1 was relabelled so that all strains within a sequence cluster were assigned to the species inferred from the positions on the tree of the type strain and other well-characterized strains. The limits of the *S. mitis *and *S. pseudopneumoniae *clusters are somewhat unclear and the strains on the flanks of this cluster are shown as white circles (uncertain species) to indicate this. Strains that were re-checked as they were outliers of species clusters, or were distinct from all other strains, are indicated by a red asterisk. B. The tree constructed from the same set of concatenated sequences using minimum evolution. C. The neighbour-joining tree with the Mitis group clusters collapsed, to show more clearly the sequence clusters within the Anginosus and Salivarius groups. The positions of the type strains and bootstrap values for the nodes are shown.

The strains within the major sequence clusters correlated sufficiently well with the species names obtained through standard taxonomic procedures, and included the type strain of the species, that each of the sequence clusters was considered to represent a species cluster. Figure 2A shows a neighbour-joining tree for all 420 strains with the strains within each cluster coloured according to their assigned species names. Figure 2C shows the same tree with the Mitis group clusters collapsed to show better the resolution of the species clusters within the Anginosus and Salivarius groups. A few strains that were on the fringes of sequence clusters were not coloured, to represent uncertainty as to the species to which they belong. The tree shown in Figure 2A was used as the MLSA reference tree for assigning a query strain to a viridans group species from its location within one of the defined species clusters.

### Comparing clustering patterns using different MLSA schemes and different tree-building methods

**Figure 3:**
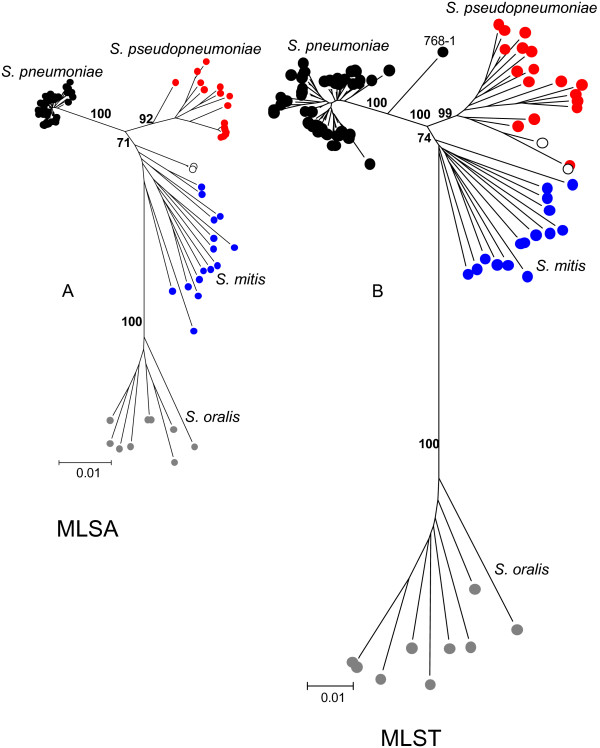
**Comparison of the clustering of strains using two different MLSA schemes**. The neighbour-joining tree obtained for a set of 93 strains of *S. pneumoniae*, *S. pseudopneumoniae*, *S. mitis *and *S. oralis *using the seven-locus MLSA scheme (A) was compared with the tree obtained from the same strains using the concatenated sequences of six of the loci used in the *S. pneumoniae *MLST scheme (B). The two trees are drawn to the same scale.

**Figure 4:**
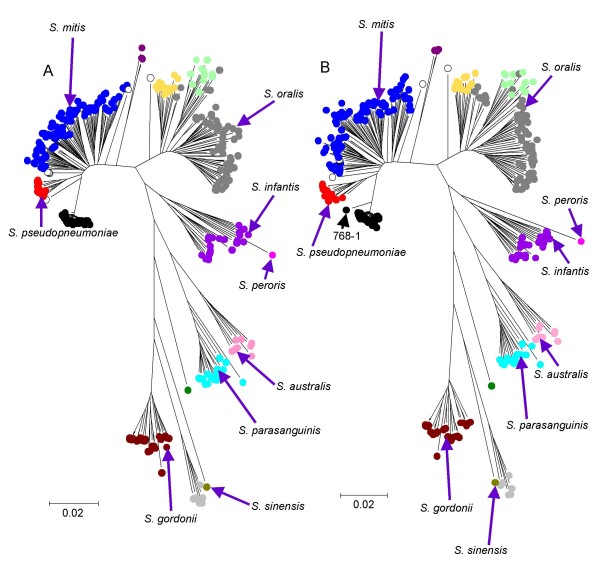
**Effect on clustering patterns of adding an additional locus (*guaA*) to the seven-locus scheme**. The *guaA *gene was successfully sequenced from 326 of the Mitis group strains and the neighbour-joining tree obtained for these strains using the seven-locus MLSA scheme (A) was compared with that obtained from the same strains by adding the *guaA *sequence to the concatenated sequences of the seven loci (B). The colour code for species clusters is as in Figure 2. The positions on the tree of the type strains are indicated.

If MLSA is to be a reliable approach to identifying species as sequence clusters, the same clusters should be obtained for the same set of strains with different sets of house-keeping genes. Figure 3 shows a comparison of the trees produced from a set of 93 strains of *S. pseudopneumoniae*, *S. pneumoniae*, *S. mitis *and *S. oralis *that were characterized by both the new seven-locus MLSA scheme and the earlier scheme that used six of the pneumococcal MLST genes [11,15]. Although these schemes used completely different house-keeping genes, the trees were remarkably similar. The genetic distances between clusters were, however, less with the new MLSA scheme than with the earlier scheme (Figure 3). There were two minor differences between the trees. Firstly, two strains on the fringes of the *S. pseudopneumoniae *cluster using the MLST loci were allied to the *S. mitis *cluster using the seven-locus MLSA scheme and were re-assigned as uncertain species. Secondly, one strain (768-1) was an outlier of the *S. pneumoniae *sequence cluster in the six-locus tree but not in the seven-locus tree; however, its *guaA *gene was unusually divergent and it was also an outlier when this gene was added to the seven-locus MLSA scheme (Figure 4B).

The clustering patterns were not greatly influenced by the tree-building algorithm. Figure 2B shows the tree produced by minimum evolution (a method based on local optimization of an initial neighbour-joining tree), compared with that obtained using neighbour-joining (comparisons were restricted to these two methods since both are computationally efficient, as required for the rapid generation of trees on-line with sequences from large number of strains). The assignment of strains to the sequence clusters was the same, and the only significant difference was a slightly more marked sub-division of the *S. mitis *group into sub-clusters using minimum evolution. As expected for sequences that are closely related, using a genetic distance correction made no difference to the clustering patterns (data not shown).

### Effect of adding an eighth gene to the MLSA scheme

To assess whether the addition of an eighth gene improved the resolution of sequence clusters (particularly of *S. mitis *and *S. pseudopneumoniae*), the 326 Mitis group strains in which the *guaA *gene could be amplified were used to produce an eight-locus tree, that could be compared with the seven-locus tree produced for all Mitis group strains (Figure 4). There were only minor differences between the eight and seven-locus trees and, excepting the location of the *S. peroris *type strain as either within, or an outlier of, the *S. infantis *cluster, none of the differences affected the resolution of the sequence clusters, and the seven-locus scheme was chosen for all further work.

### Identification of potential new viridans group species

The MLSA scheme should be able to identify possible new species as groups of strains, or individual strains, which clearly do not fall within any of the known species clusters. We firstly checked whether strains that did not fall into known species clusters, or were outliers of species clusters, could be the result of using DNA from mixed cultures. In all cases, further sub-culturing of these strains (labelled in Figure 2A) and re-sequencing of the seven loci gave the same results as obtained initially, ruling out the possibility that the position on the concatenated tree of these strains was due to the use of DNA from mixed cultures.

One example of an unidentified cluster is provided by four closely related strains (assigned by MK as *S. mitis*) that arose as a distinct lineage from the branch separating the *S. mitis *and *S. oralis *clusters (unknown A in Figure 2). These four strains were isolated from members of two families included in a study of the clonal diversity of *S. mitis *within individuals [40]. Although the strains cannot be distinguished from *S. mitis *by phenotypic analysis, they may constitute a separate species.

**Figure 5:**
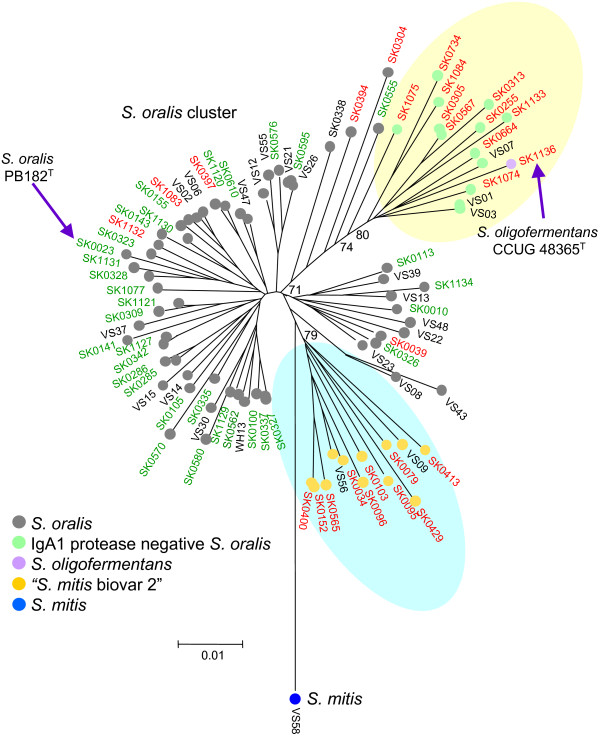
**Phenotypically distinct sub-clusters within the *S. oralis *species cluster**. The region of the neighbour-joining tree that includes strains within the *S. oralis *species cluster is shown in more detail. The positions on the tree of the type strains are indicated. The sub-cluster of phenotypically distinct strains that are arginine hydrolysis and α-maltosidase positive, and which express the Lancefield group K cell wall carbohydrate antigen, is indicated (highlighted in blue). A further subgroup, which included the *S. oligofermentans *type strain (SK1136), consisting exclusively of IgA protease-negative strains is also indicated (highlighted in yellow). Bootstrap values for relevant nodes are shown. Green strain names indicate IgA1 protease-positive strains whereas in red they are IgA1 protease negative. The IgA1 protease status of a few strains (black strain names) was unknown.

Likewise, the two major clusters that include the type strains of *S. mitis *and *S. oralis *both include several sub-clusters, which may warrant recognition as separate species. As an example, 10 strains previously assigned to '*S. mitis *biovar 2' [25], formed a sub-cluster (including strains SK34, SK79, SK96 and others) within the major *S. oralis *cluster (Figure 5), in agreement with recent observations [18]. These strains are phenotypically distinct from the remaining part of the *S. oralis *cluster by being arginine hydrolysis and α-maltosidase positive, and by expressing the Lancefield group K cell wall carbohydrate antigen (data not shown). In addition, all strains of the '*S. mitis *biovar 2' sub-cluster lacked IgA1 protease activity (Figure 5).

Another sub-cluster within the major *S. oralis *cluster was also composed exclusively of strains that lacked IgA1 protease activity, while only six of the 59 *S. oralis *strains that were not in the above two sub-clusters were IgA1 protease negative (Figure 5). Surprisingly, the latter IgA1 protease-negative sub-cluster included the designated type strain of *S. oligofermentans*, in spite of its significantly different 16S rRNA sequences [41].

Apart from these examples, none of the other sub-clusters identified within the *S. mitis *and *S. oralis *clusters had distinguishing phenotypic properties, and their potential recognition as separate taxa should await further expansion of the eMLSA database and comprehensive phenotypic characterization of strains. A single strain (unknown B) was located on a long branch on the reference tree between *S. agalactiae *and the species clusters of the Salivarius group (Figure 2C) and its taxonomic status is unclear. However, from BLAST comparisons of the house-keeping genes of this strain to the nucleotide sequence databases this strain is almost certainly a member of the Bovis group streptococci.

### Differences in diversity of species clusters

There were substantial differences in the extent of diversity within the species clusters and in their patterns of branching. For example, *S. pneumoniae *strains formed a tight sequence cluster on the tree and the average within-species diversity was only 0.3%, whereas the average diversity within several other clusters, including those of *S. anginosus*, *S. australis*, *S. infantis*, *S. mitis *and *S. oralis*, was 12 to 16 times greater (Table 2). Additionally, unlike *S. pneumoniae *and *S. pyogenes*, where there is a branching structure within the species clusters, with some strains being almost identical and others being more distantly related, the other species clusters typically included strains that were relatively distantly related to each other (for example, the *S. infantis *and *S. mitis *clusters), as implied by the long branch lengths from each strain in a cluster to its node (Figure 2A).

**Table 2:** Average nucleotide sequence diversity within species clusters

Species cluster	Average diversity
*S. pneumoniae*	0.003
*S. vestibularis*	0.006
*S. thermophilus*	0.009
*S. constellatus*	0.009
*S. pseudopneumoniae*	0.014
*S. pyogenes*	0.018
*S. salivarius*	0.020
*S. cristatus*	0.022
*S. intermedius*	0.027
*S. gordonii*	0.032
*S. sanguinis*	0.033
*S. parasanguinis*	0.034
*S. anginosus*	0.036
*S. oralis*	0.036
*S. oralis *– IgA1 protease negative	0.036
*S. mitis*	0.038
"*S. mitis *biovar 2"	0.039
Unknown A	0.043
*S. australis*	0.046
*S. infantis*	0.050

Species are listed in ascending order of intra-species sequence diversity.

### Clustering patterns obtained using single gene trees and identification of interspecies imports

**Figure 6:**
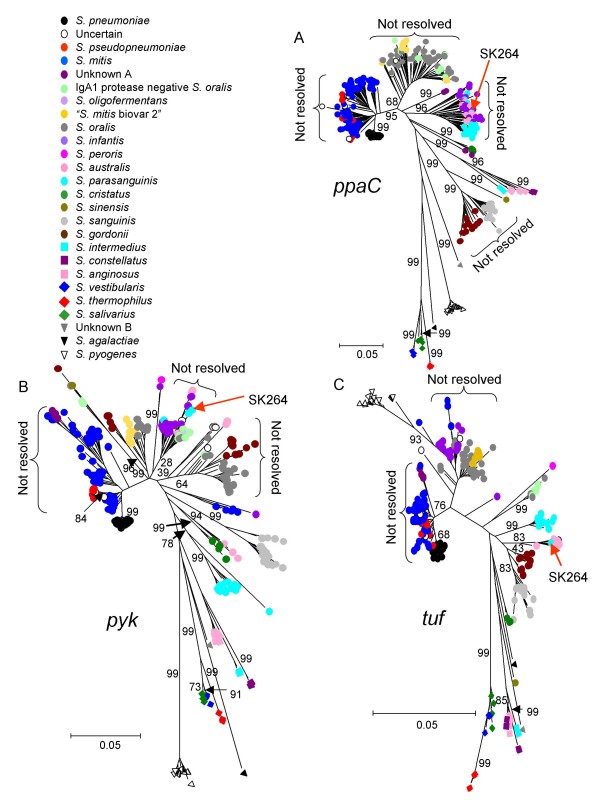
**Examples of individual gene trees produced from the sequences of all 420 strains**. A-C shows the *ppaC, pyk and tuf *gene trees, which each fail to resolve some of the species clusters. Strain SK264 (assigned as *S. parasanguinis*; labelled in Figure 2A) was used as the query strain in Figures 7 and 8. The *ppaC *allele of this strain is assigned as resident compatible as it falls within an unresolved cluster that includes the other *S. parasanguinis *sequences. For both *pyk *and *tuf*, the sequences from SK264 fall within clusters that are well resolved (bootstrap values of ≥ 80%) from the cluster that includes all (or the great majority) of the other *S. parasanguinis *sequences and are assigned as foreign alleles. For *pyk *the source of the foreign allele is unclear as its sequence falls within a cluster that includes those from several species. For *tuf*, the sequence appears to have been introduced from an *S. australis *strain. The major unresolved clusters in the trees are indicated.

Neighbour-joining trees were constructed from the sequences of the seven individual genes from all strains. These gene trees failed to resolve the species clusters obtained using the concatenated sequences and all individual trees showed anomalous clustering of some strains (Figure 6; Additional file 1). For example, *S. mitis *and *S. pseudopneumoniae *could only be resolved on the *map *and *pyk *gene trees (Figure 6B). Similarly, strains of *S. infantis *and *S. australis *could not be resolved on the *sodA *gene tree, and strains of neither of these species could be resolved from each other, or from *S. parasanguinis*, on the *ppaC *tree (Figure 6A).

Approximately 96% of strains assigned to a particular species by MLSA had alleles at all seven loci that were most similar to those of other strains of that species and, in individual gene trees, the sequences clustered with those of other strains of the species. However, 16 of the viridans group strains (4%) had alleles that were considered to be foreign (see Methods for criteria used to assign alleles as foreign). Table 3 shows the putative origins for each of the seven alleles in these 16 strains.

**Table 3:** Strains possessing alleles from other species

**Species**	**Strain**	***map***	***pfl***	***ppaC***	***pyk***	***rpoB***	***sodA***	***tuf***
Unknown A	SK638	resident*	resident	resident	resident	resident	*S. mitis*	resident
*S. gordonii*	SK121	resident	resident	resident	uncertain**	resident	resident	resident
IgA1 protease negative *S. oralis*	SK313	resident	resident	resident	uncertain	resident	resident	resident
IgA1 protease negative *S. oralis*	SK734	resident	resident	resident	resident	resident	*S. australis/S. infantis*	resident
IgA1 protease negative *S. oralis*	SK1084	resident	resident	resident	resident	resident	*S. australis/S. infantis*	resident
IgA1 protease negative *S. oralis*	SK1133	resident	resident	resident	uncertain	resident	resident	resident
*S. oralis*	SK304	resident	resident	resident	uncertain	resident	resident	IgA1 protease negative *S. oralis*
*S. oralis*	SK309	resident	resident	resident	resident	resident	*S. australis/S. infantis*	resident
*S. oralis*	SK394	resident	resident	resident	resident	resident	resident	IgA1 protease negative *S. oralis*
*S. oralis*	SK555	resident	resident	resident	resident	resident	resident	IgA1 protease negative *S. oralis*
*S. oralis*	VS37	resident	resident	resident	resident	resident	*S. australis/S. infantis*	resident
*S. parasanguinis*	SK236	resident	resident	resident	*S. infantis/S. oralis*/IgA1 protease negative *S. oralis*	resident	resident	resident
*S. parasanguinis*	SK264	resident	resident	resident	*S. infantis/S. oralis*/IgA1 protease negative *S. oralis*	resident	resident	*S. australis*
*S. parasanguinis*	SK438	resident	resident	resident	*S. infantis*/*S. oralis*/IgA1 protease negative *S. oralis*	resident	resident	resident
*S. pseudopneumoniae*	IOPR1711	resident	resident	resident	resident	*S. pneumoniae*	resident	resident
*S. thermophilus*	19258	resident	uncertain	resident	resident	resident	resident	resident

Foreign alleles are underlined. *Resident or resident compatible allele. **Foreign allele but donor species uncertain.

### Assigning strains to species via the internet

**Figure 7:**
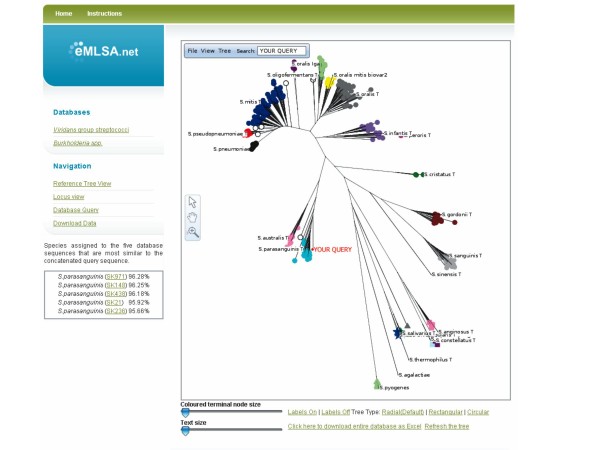
**Species assignment on the internet**. The eMLSA.net page returned after entering the seven gene sequences of a query strain and requesting a species assignment. The species assigned to the five most closely matching concatenated sequences are returned (left) along with an unrooted neighbour-joining radial tree indicating the position of the query strain. In this case, the query strain (SK264) is assigned as *S. parasanguinis *as the five most similar concatenated sequences are all from this species and the query strain falls within the *S. parasanguinis *species cluster on the tree.

The main features of the eMLSA.net website are described in the Methods section. Figure 7 is a screenshot from the species assignment window of  , showing the position of a query strain (*S. parasanguinis *SK264) on the reference tree, and the list of species assignments of the five strains in the database with the most similar concatenated sequences, and their sequence similarity to the query sequence. In this example the query strain is assigned as *S. parasanguinis *since the concatenated sequence of the seven MLSA loci clusters within this species cluster, and the top five most similar concatenated sequences are from strains assigned to this species.

**Figure 8:**
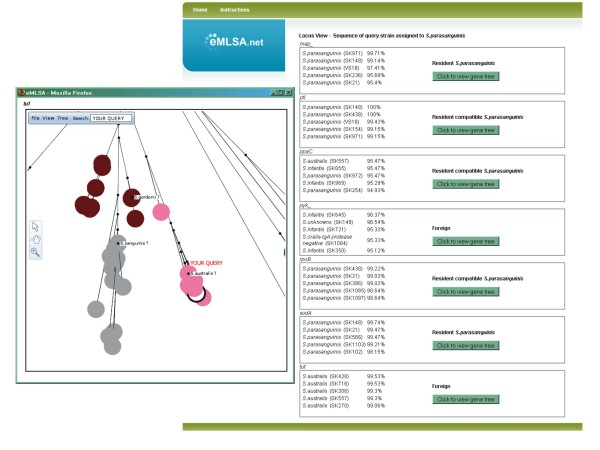
**Online assignment of alleles as resident or foreign**. Having assigned a query strain to a species (Figure 7), the locus view page shows the assignment of each of the seven individual sequences as resident to that species (or compatible with being resident) or foreign. In the example, the sequences of five of the genes from strain SK264 are assigned by eMLSA.net as resident (that is, *S. parasanguinis*) or resident compatible, but the *pyk *and *tuf *genes are assigned as foreign. The locus view page allows the individual gene trees (in this case, the *tuf *tree) to be displayed to explore why the algorithm considers the sequences of an allele of a query strain to be resident, resident compatible or foreign (see Figure 6 for details). The colour codes for species clusters are as in Figure 2.

Figure 8 shows a screenshot of the resident, resident compatible or foreign status assigned by eMLSA.net to each of the seven individual alleles of the above query strain. In this case, five of the individual loci are most similar to sequences from *S. parasanguinis *(resident alleles) or are resident compatible (for example, *ppaC*; Figure 6A). However, the *pyk *gene is within a cluster that is well resolved from the main *S. parasanguinis *cluster and which includes *S. infantis*, *S. australis *and *S. cristatus *(Figure 6B), and the *tuf *gene clusters within the well-resolved *S. australis *cluster (Figure 6C). These two alleles are therefore assigned by eMLSA.net as foreign, which may explain why this strain is an outlier within the *S. parasanguinis *cluster (Figure 2A).

## Discussion

The aim of this paper was to produce a generic electronic taxonomy website and software that can be used for the assignment of bacterial strains to species via the internet and to develop a comprehensive dataset that can form a framework for establishing a more satisfactory approach to prokaryotic taxonomy. The viridans group streptococci were chosen as an exemplar of electronic taxonomy using the MLSA approach since their taxonomy by traditional means has been problematic, with poorly defined species, frequent revisions and changes of species names [24].

The seven-locus MLSA scheme resolved sequence clusters that were broadly consistent with those expected from the species assignments provided by standard taxonomic methods. Importantly, except for *S. peroris *and *S. oligofermentans*, the sequence clusters included the type strain of only a single species which, in a formal taxonomic sense, defines these as species clusters. The location of the *S. peroris *type strain on a long branch within the *S. infantis *cluster is consistent with the close relationship between these species in the tree based on four loci described by Hoshino et al [16], and with % DNA-DNA hybridization values as high as 62% between some strains of *S. infantis *and *S. peroris *[30]. The lack of any other strains assigned to the latter species precluded any further exploration of the validity of the distinction between these two very similar species, but the species status of *S. peroris *needs to be re-assessed.

The type strain of *S. oligofermentans *[41] was within the *S. oralis *species cluster. Similarly, six of the seven individual loci clustered with *S. oralis *strains; the other locus (*sodA*) clustered with *S. mitis*. Sequences of three of the MLSA genes (*sodA*; accession number DQ232554, *tuf*; EU156926 and *rpoB*; DQ232508) from the same strain of *S. oligofermentans *have been deposited in the nucleotide sequence databases. These genes have sequences identical to those we obtained, eliminating the possibility of any errors in our data on this strain, and the species status of *S. oligofermentans *also needs to be re-examined.

Otherwise, the major anomaly was the apparent difficulty that taxonomists have in recognizing strains of *S. mitis*; strains assigned on standard taxonomic grounds to this species were found among several clusters, although the major cluster included the *S. mitis *type strain. This problem is largely resolved by the recognition that '*S. mitis *biovar 2' strains are a sub-cluster within the major *S. oralis *cluster, as also suggested by Kilian et al [18].

MLSA schemes using different sets of house-keeping genes should give the same clusters and this requirement was fulfilled, although the resolution between *S. pseudopneumoniae *and *S. mitis *was less clear using the concatenated sequences of the seven genes than with the six MLST genes. The poorer resolution may be a consequence of the method used to ensure that the gene fragments could be amplified from all Mitis, Anginosus and Salivarius group species (see Methods), which may favour genes that evolve more slowly than the average house-keeping gene. This was apparently the case as the genetic distances between clusters was clearly less with the new MLSA scheme compared with the earlier scheme (Figure 3). However, the new scheme is favoured as it allows a much wider group of species to be studied, allowing strains to be assigned via the internet to all Mitis, Anginosus and Salivarius group species (and to some other non-viridans streptococci including *S. agalactiae *and *S. pyogenes*) using the initial database of 420 strains. The concatenated sequences of some of these species differed at over 20% of nucleotides and it is likely that the degenerate primers will amplify the seven loci from other closely related streptococcal species, allowing the MLSA scheme to assign strains to these additional species.

As new strains are added to the database, electronic taxonomy allows the identification of new sequence clusters that can be assigned as new species. Additionally, the mapping of phenotypic characteristics onto sequence clusters provides an important way of exploring the consistency of taxonomic assignments. In some cases this may produce evidence supporting the splitting of a cluster into more than one species. This was evident among the *S. oralis *species cluster where one sub-cluster, corresponding to '*S. mitis *biovar 2', had clearly distinctive biochemical properties, which may be an argument for recognizing it as a separate species.

The individual gene trees failed to identify the sequence clusters that were resolved with the concatenated sequences, a phenomenon observed previously in an MLSA study of *S. pneumoniae*, *S. pseudopneumoniae*, *S. mitis *and *S. oralis *[11,15]. The most likely reasons for the differences in the individual gene trees are either that there were insufficient informative sites to resolve some species using a single gene fragment, or that a history of interspecies recombination has distorted the relationships between species clusters. There was, however, sufficient signal in the individual gene trees to resolve the species clusters using the concatenated sequences of all seven genes.

Only 16 strains clearly had foreign alleles, which presumably had been introduced by interspecies recombination from another viridans group species. However, the approach used would not detect recombination between very similar species that are not resolved on individual gene trees (which are likely to be the most common events; see below) and, together with the conservative high bootstrap value (≥ 80%) used to define clusters as well resolved, this may have lead to a considerable underestimate of the extent of inter-species recombination within this group. The efficiency of recombination falls off as the logarithm of the sequence divergence between the donor and recipient strains [42] and, as expected from this relationship, where the source of alleles could be clearly assigned, most foreign alleles were from donor species that clustered on the concatenated tree close to the recipient species. For example, the source of foreign alleles in *S. pseudopneumoniae *was *S. pneumoniae*, and for *S. oralis *it was *S. australis/S. infantis *andIgA1 protease-negative S. *oralis*.

Interspecies recombination could lead to a strain clustering on the tree away from other strains of the species if the recombinational replacement is from an unusually divergent donor, or there is more than one locus that has received a recombinational replacement. This phenomenon has been found with *Salmonella enterica *var Typhi strains, where six of the genes used in MLSA clustered within those of other strains of *S. enterica*, whereas the *purE *sequence was about 6% divergent such that, using the concatenated sequences, the Typhi strains clustered slightly apart from the *S. enterica *cluster [43]. This potential problem can be recognized using the MLSA website by examination of the individual gene trees and the resident or foreign status of the alleles (and the percentage divergence) in strains that are outliers of known species clusters.

How do the results described here inform the way bacterial species should be defined? Firstly, the patterns of clustering observed in the viridans group have relevance to any method that attempts to define bacterial species using fixed cut-off values, whether these are based on percent DNA-DNA hybridization [9] or average percent nucleotide differences between all shared genes [44]. The very different levels of diversity among the different species clusters (Table 2) highlight the difficulties in defining bacterial species using a fixed cut-off value. The advantage of electronic taxonomy is that species clusters can be identified and circumscribed by visual inspection of the tree, without regard to preconceptions about the extent of diversity a bacterial species should have. The diversity of genotypes within some species of this group, and recombination among similar species, could make it unlikely that there will be a single phenotypic characteristic, common to all strains of a species but not to those of other similar species, which can be used to recognize each viridans group species. The requirement for a defining phenotypic characteristic for the official recognition of new species can in any case be questioned as outmoded and unnecessary if these can be identified simply by their clustering on a tree using MLSA [11]. Finally, as shown by the unexpected clustering of the *S. oligofermentans *and *S. peroris *type strains, the results highlight how MLSA can evaluate the taxonomic status and validity of proposed or accepted new species and can identify taxonomic anomalies.

The number of house-keeping loci needed for electronic taxonomy using MLSA is not yet clear, and resolution between species clusters should improve with increasing numbers of loci. However, in practice, the number will be a balance between that needed for good resolution of species clusters and that which allows the generation of large databases and the uptake of the method by taxonomic laboratories. The number is likely to vary depending on the extent of sequence diversity within the group and the extent of interspecies recombination. In groups of species where recombination is very rare, resolution between clusters will depend on the number of polymorphic sites, and the aim of MLSA is to use sufficient genes to obtain enough polymorphic sites to adequately resolve species clusters. For such highly clonal species three or four house-keeping genes may be adequate to resolve clusters. In groups where interspecies recombination occurs, multiple genes are required both to provide adequate numbers of polymorphic sites and to overcome the distorting effects of interspecies recombination at individual loci, by extracting adequate taxonomic signal from the weak signal present in each of the individual genes. Seven-locus MLSA schemes appear to be adequate for resolving closely related species that undergo relatively frequent recombination, such as the viridans streptococci.

It is tempting with a bacterial group that has been well studied to select for MLSA schemes those genes for which the individual trees give the best approximation of the expected relationships among strains. However, for a general approach to discerning clustering patterns this is unwise, as the expected clusters will not be known when poorly studied bacteria are examined. Initial MLSA studies should therefore use the concatenated sequences of all selected genes to build a tree to identify the sequence clusters. It is advisable when developing MLSA for a poorly studied group of species to select more genes than are needed for the final MLSA scheme (for example, eight or nine genes for a final seven-gene scheme), so that the least informative can be eliminated from the final scheme. The best gene(s) to eliminate can be identified by using the tree based on the concatenated sequences of all genes as the reference tree, and examining the maximum likelihood of each of the single gene trees as a fit to the reference tree, eliminating those with the lowest likelihood.

Although we have used the term electronic taxonomy, the assignment of strains to known species and the identification and acceptance of new species cannot realistically be automated as it requires the experience, knowledge and judgement of taxonomists. The assignment of species using MLSA for electronic taxonomy should therefore be a community activity, in which those interested in a particular taxonomic group can share their experience and knowledge to provide a consensual approach to deciding whether new sequence clusters should be assigned as new species. As with the MLST databases, eMLSA.net is an actively curated open-access website and submissions of MLSA data on further strains of those species that currently are represented by only a small number of strains will help to circumscribe these species. Similarly, the submission of strains that are difficult to speciate by standard methods may identify new sequence clusters, and submission of sets of strains of other closely related streptococcal species will expand the breadth of streptococcal species that can be assigned to species clusters. Furthermore, those who consider they have identified, using standard taxonomic methods, a new species within or closely related to the viridans group streptococci can perform MLSA and submit the strain(s) to the database to see whether it matches any already in the database or whether, once submitted, new strains that cluster with that of the proposed new species appear as the database grows. Developers of MLSA schemes for other closely related bacterial (or other microbial) species are encouraged to host their data at  to take advantage of the generic analysis software and to provide a single portal for the electronic taxonomy of many groups of microbial species. A site for the on-line speciation of vibrios is also available , although at present it only identifies the most similar species to a query strain using BLAST, rather than generating the more informative sequence clusters [22].

## Conclusion

The identification of species by electronic taxonomy using the MLSA approach provides a way of identifying species that is as least as valid as any other current taxonomic approach. The clustering patterns from MLSA provide a framework for species definition that can be further validated or revised by mapping on additional phenotypic, ecological or genomic data. Finally, this approach to electronic taxonomy, and our generic MLSA website, are not restricted to identifying and circumscribing bacterial species and could be applied to the Archaea and some eukaryotic microorganisms.

## Methods

### Bacterial strains

The Mitis group streptococci studied here included strains (numbers of strains are shown in parentheses) assigned by standard taxonomic procedures (or, subsequently, from their clustering patterns) as *S. australis *(10), *S. cristatus *(5), *S. gordonii *(28), *S. infantis *(24), *S. mitis *(88), *S. oligofermentans *(1), *S. oralis *(84), *S. parasanguinis *(25), *S. peroris *(1), *S. pneumoniae *(51), *S. pseudopneumoniae *(20), *S. sanguinis *(25) and *S. sinensis *(1). Strains of *S. anginosus *(7), *S. constellatus *(4) and *S. intermedius *(2) (Anginosus group streptococci), *S. salivarius *(4) and *S. vestibularis *(4) (Salivarius group streptococci) and *S. pyogenes *(17) (Pyogenic group streptococci) and *S. agalactiae *(1) were also studied. Sequences of the MLSA loci of the third member of the Salivarius group, *S. thermophilus*, were obtained from the type strain (ATCC 19258^T^) and from the genome sequences of strains LMD-9, CNRZ1066 and LMG 18311 (GenBank: CP000421, CP000024 and CP000023). Two strains of *S. pseudopneumoniae *(one of which is the type strain ATCC BAA-960^T^) were from the study of Arbique et al [45], who proposed this new Mitis group species. Another 21 strains were considered to be this species as they (and the above two strains) formed a well-resolved sequence cluster using the MLSA scheme based on six MLST loci [11]. Three of these were subsequently re-assigned as uncertain species (see Results).

The type strains of all of the viridans species, except *S. pneumoniae*, were included in the strain collection. Strains assigned, or tentatively assigned, to the above viridans species (except *S. pneumoniae *and *S. pseudopneumoniae*) were selected from the collection of MK and were sent (in most cases without the species name assigned by the laboratory of MK) to Imperial College London for MLSA. In addition, a number of strains that were difficult to assign to the recognized species were included, as well as 52 viridans streptococci recovered in a UK hospital from infections in neutropenic patients [46]. The *S. pyogenes *and *S. pneumoniae *strains were from the collections of WPH and BGS. In total there were 420 sets of sequences, corresponding to those obtained from 399 viridans group strains (plus three extracted from *S. thermophilus *genome sequences), 17 obtained from *S. pyogenes *strains and one from *S. agalactiae*. Details of all strains are available as Additional file 2.

The sequences of the seven MLSA loci were also obtained from the complete genome sequences of *S. mutans *UA159 (GenBank:AE014133), *S. uberis *0140J (Sanger Institute website; ), *S. equi *4047 (Sanger Institute website) and *S. suis *P1/7 (Sanger Institute website). The positions of the latter species on the reference tree are not shown in this paper as we have not examined whether all seven MLSA genes can be amplified from these species with the viridans group primer set, but there is an option to include these strains on the reference tree generated at the eMLSA.net website.

### Assignment of strains to viridans species using standard taxonomic methods

The strains from the laboratory of MK were initially identified over a period of 20 years according to the current taxonomic status by a combination of extensive phenotypic analysis, sequencing of 16S rRNA and sequencing of selected house-keeping genes [16,18,25]. Serotypable *S. pneumoniae *strains were identified by standard methods; non-typable presumptive pneumococci were assigned to either *S. pneumoniae *or *S. pseudopneumoniae *as described previously [11].

### Multilocus sequence analysis of viridans group streptococci

Suitable loci for the discrimination of species clusters must be present in all strains of the species under study, and should be sufficiently conserved that a fragment of each locus can readily be amplified from each species using a single set of primers, but sufficiently diverse that they are useful in resolving species clusters. Two of the loci used in the new MLSA scheme (*rpoB *and *sodA*) were included in the study of Hoshino et al [16]. To select additional house-keeping loci, we used the multi-genome homology comparison tool available through the Comprehensive Microbial Resource (CMR; ) to search for proteins present in all available streptococcal genomes that had at least 80% amino acid sequence identity. This retrieved 138 proteins and, from those that had house-keeping functions, the corresponding genes were selected at random and examined for their suitability as candidate MLSA loci. An alignment was produced of the sequences of each candidate locus from *S. agalactiae *NEM3 (GenBank:AL732656), *S. mitis *NCTC 12261 (available at J Craig Venter Institute website; ), *S. mutans *UA159 (GenBank:AE014133), *S. pneumoniae *TIGR4 (GenBank:AE005672), *S. pyogenes *MGAS10394 (GenBank:CP000003), *S. thermophilus *LMD-9 (GenBank:CP000421) and *Lactococcus lactis *II1403 (GenBank:AE005176). Degenerate primers were designed for each selected locus, based on stretches of six amino acids that were conserved in all these species, which would allow an internal gene fragment greater than about 350 bp to be fully sequenced on both strands, and in which no indels were found in any of these genomes.

The pairs of PCR primers were each checked for their ability to amplify the correct gene fragment from a diverse subset of the collection of viridans group streptococci. Those primers that failed to amplify the correct fragment from all of the subset of strains were replaced with primers for amplifying a fragment of another of the conserved house-keeping genes, until a set of eight genes and PCR primers were obtained that allowed amplification of an internal gene fragment of each gene from the subset of strains. On characterizing the full strain set it was found that the primers for *guaA *(and two alternative pairs of primers) failed to amplify the fragment from a small number of strains and this gene was dropped from the final MLSA scheme. The genes, gene products, primer sequences, length of the sequences used in the MLSA scheme, and the annealing temperatures for amplification are shown in Table 1.

The gene fragments were amplified by PCR (30 cycles) using, for each gene fragment, a single annealing temperature for all strains (Table 1), and were sequenced on both strands, using the primers for the initial amplification, with an ABI 3700 or ABI 3730xl DNA analyzer. The sequences were aligned and trimmed to defined start and end positions using MEGA version 4 [47]. The trimmed sequences of the seven gene fragments from strains of each species can be found at . The sequences of the seven gene fragments from each strain were joined in-frame, in the order *map-pfl-ppaC*-*pyk-rpoB*-*sodA*-*tuf*, to generate a single 3063 bp concatenated sequence. Unrooted individual gene trees, and trees obtained using the concatenated sequences, were generated by neighbour-joining or minimum evolution from the proportions of sequence differences between all strains using MEGA version 4 [47]. The robustness of the nodes was evaluated by bootstrapping (1000 replicates). All of the sequences (including the concatenated sequences and the sequences of each locus) from the 417 strains can be downloaded from .

### Electronic taxonomy website

A generic web application for electronic taxonomy was developed at . This open-access site serves three main functions. It provides a database of all characterized strains of a group of similar species (in this case, all of the 420 streptococcal strains characterized by MLSA), to which new strains can be added by the user community. Secondly, it allows the species assignment of query strains and, thirdly, it allows the identification of alleles that appear to have been introduced into the query strain from a closely related species.

The database (available to download at ) consists of a table containing strain information including, for each entry, the strain name; the species name assigned by MLSA and by standard taxonomic methods (a comment field provides the basis for these species assignments; another field allows the entry of any unusual features of the strain compared with typical strains of the assigned species); the country of origin, date of isolation and source of the strain (nasopharynx, blood, etc); whether it is a type strain; the names and addresses of the laboratories or culture collections that hold the strain; the contact details of the person who submitted the strain data; and any publication relating to the properties or taxonomic status of the strain. Further database tables hold the sequences that correspond to the alleles at each locus.

### On-line species assignments

To observe the patterns of clustering of the strains in the database, and to show the species cluster into which a user's strain falls, the seven edited and trimmed sequences of a query strain are cut and pasted into individual text boxes. Sequences are checked for the correct length for the locus and the absence of any characters other than A, C, G and T; the submitter is alerted if any entered sequence is of non-standard length or contains unexpected characters. If the sequences are all of the expected length, they are concatenated in-frame, and the query sequence is added to the concatenated sequences of all strains in the MLSA database. To construct the tree, a module of MEGA was used (kindly provided by Dr Sudhir Kumar, Arizona State University), which takes the concatenated sequences and generates in Newick format either a neighbour-joining or minimum evolution tree, incorporating options for a range of genetic distance corrections and for bootstrap re-sampling. If the sequences have insertions or deletions (indels), a modification of the software is used that generates an alignment of the concatenated sequences, which is used by the MEGA module to produce the tree. A module of Phylowidget (; [48]) developed using the visual programming language Processing , provides easy communication between the tree diagram and the underlying database. Phylowidget is embedded within the website and is passed the Newick file generated by the MEGA module for display as a radial tree (the tree can also be displayed in the standard rectangular format or in circular format). The position of the query strain is highlighted on the tree and, using the intuitive click-drag-zoom interface, the tree can be explored further. Individual gene trees can also be constructed by the same method. A standard database search facility is available allowing details of any strain or set of strains to be viewed, and a search box on the reference tree allows the position of any strain to be highlighted on the tree.

A species assignment can be proposed if the concatenated sequence of the strain falls within a known species cluster, and it can be assigned as an unknown species if it falls within a cluster with no assigned species name, or as a unique divergent strain if it clusters apart from any of the sequence clusters. In addition, a list of the species names assigned to the five concatenated sequences in the database that are most similar to the concatenated query sequence is shown, together with the % sequence similarity values to these strains (an alert shows if there are less than five strains of this species in the database).

The tree produced on-line must be the same for all users of the website and only a single tree-building method is employed within eMLSA.net and, as the tree is to discern clustering patterns rather than phylogeny, an unrooted tree is generated and is displayed by default in radiation format, although other tree display formats can be selected by the user. The default settings for the trees produced for a particular MLSA scheme (for example, minimum evolution or neighbour-joining), can be changed by the curator of the scheme to any of those provided by the MEGA module, and one of a number of different genetic distance corrections can be applied. All of the concatenated sequences can be downloaded from the website in MEGA format to allow other types of tree to be constructed off-line, using MEGA4 or alternative tree-building packages.

### On-line assignment of source of individual alleles

To assign the alleles at each locus in a strain as 'resident', 'resident compatible' or 'foreign', the sequence at each locus of a query strain that has been assigned to species A is compared with all other sequences at that locus, and the species assigned to the strains with the five most similar sequences are displayed (an alert shows if there are less than five strains of the latter species in the database). If the most similar sequences are all from strains of species A, the sequence at that locus is defined as a resident allele. However, if the best matches are sequences from strains assigned to another species, the allele is considered to be foreign, as long as species A and the species cluster assigned for that allele are well resolved on the individual gene tree. Resident compatible is used where the allele sequence is within a cluster of two or more species (including species A) that are not resolved on the gene tree. Resolution and lack of resolution between clusters are guided by the bootstrap values for the nodes on the individual gene trees separating the relevant species. A cluster is defined as resolved if the bootstrap value of the node separating it from neighbouring clusters is ≥ 80%.

To ensure that the assignment of alleles as foreign is dependent on good resolution on the gene tree, a look-up table was generated for each locus, which lists those species that could not be resolved on the gene tree for that locus. For example, only two of the individual gene trees (*map *and *pyk*) could resolve *S. mitis *and *S. pseudopneumoniae *strains (see Additional file 1). Thus, if the concatenated sequence of a particular strain was assigned to the *S. mitis *cluster, but it had its five closest matches at *rpoB *with *S. pseudopneumoniae *strains (or a mixture of *S. mitis *and *S. pseudopneumoniae *strains), this would not be scored as a foreign *rpoB *allele within the *S. mitis *strain, as the look-up table indicates that the *rpoB *tree cannot resolve *S. mitis *and *S. pseudopneumoniae*. This result is therefore consistent with the *S. mitis rpoB *allele being a resident allele (resident compatible). Where the best matches are not from species A, but are from two species that are both well resolved from species A (but not from each other), the allele at that locus is assigned as foreign, and the source is shown to be one or other of the latter two species. The website then returns the predicted resident, resident compatible or foreign status of each of the sequences at the seven loci for the query strain (or for any strain in the database).

For each locus, the website also shows the % sequence divergence of the query sequence from the best-matching sequences. These values identify sequences that are highly divergent from other alleles of the same species, as one or more divergent alleles could result in anomalous clustering of the concatenated sequence of the strain.

### Strain submission to eMLSA.net

Submission to the eMLSA.net curator of the details of new strains, with their sequences at the seven loci and the original sequencer trace files, and proposed species assignment, is strongly encouraged to build up the viridans group electronic taxonomy database. After checking the sequences for quality and correct trimming, and the proposed species assignment, each new strain and the strain details are added to the database. Submission of viridans group strains that do not fall within the existing sequence clusters are particularly encouraged to allow the identification of potential new species within this group.

## Authors' contributions

CJB carried out the experimental work, DMA developed the eMLSA website, GEJ produced a version of Phylowidget for the eMLSA website, and MK provided strains and expertise in streptococcal taxonomy. WPH and BGS conceived the project. All authors contributed to the writing of the paper. All authors have read and approved the final manuscript.

## Supplementary Material

Additional file 1 **Supplementary figure.** Individual gene trees. (A-C) Neighbour-joining trees were produced for each MLSA gene using the different sequences at each locus present among the 420 strains. The colour codes for species clusters are as in Figure 2. Click here for file

Additional file 2 **Supplementary table.** Database of strain Click here for file
