# Supplementary material for: Assigning strains to bacterial species via the internet
Source: BMC Biol. 2009 Jan 26;7:3. doi: 10.1186/1741-7007-7-3 (PMC2636762; doi:10.1186/1741-7007-7-3)
Supplement: Additional file 1 — Supplementary figure. Individual gene trees. (A-C) Neighbour-joining trees were produced for each MLSA gene using the different sequences at each locus present among the 420 strains. The colour codes for species clusters are as in Figure 2. [file 1741-7007-7-3-S1.ppt]

## Slide 1
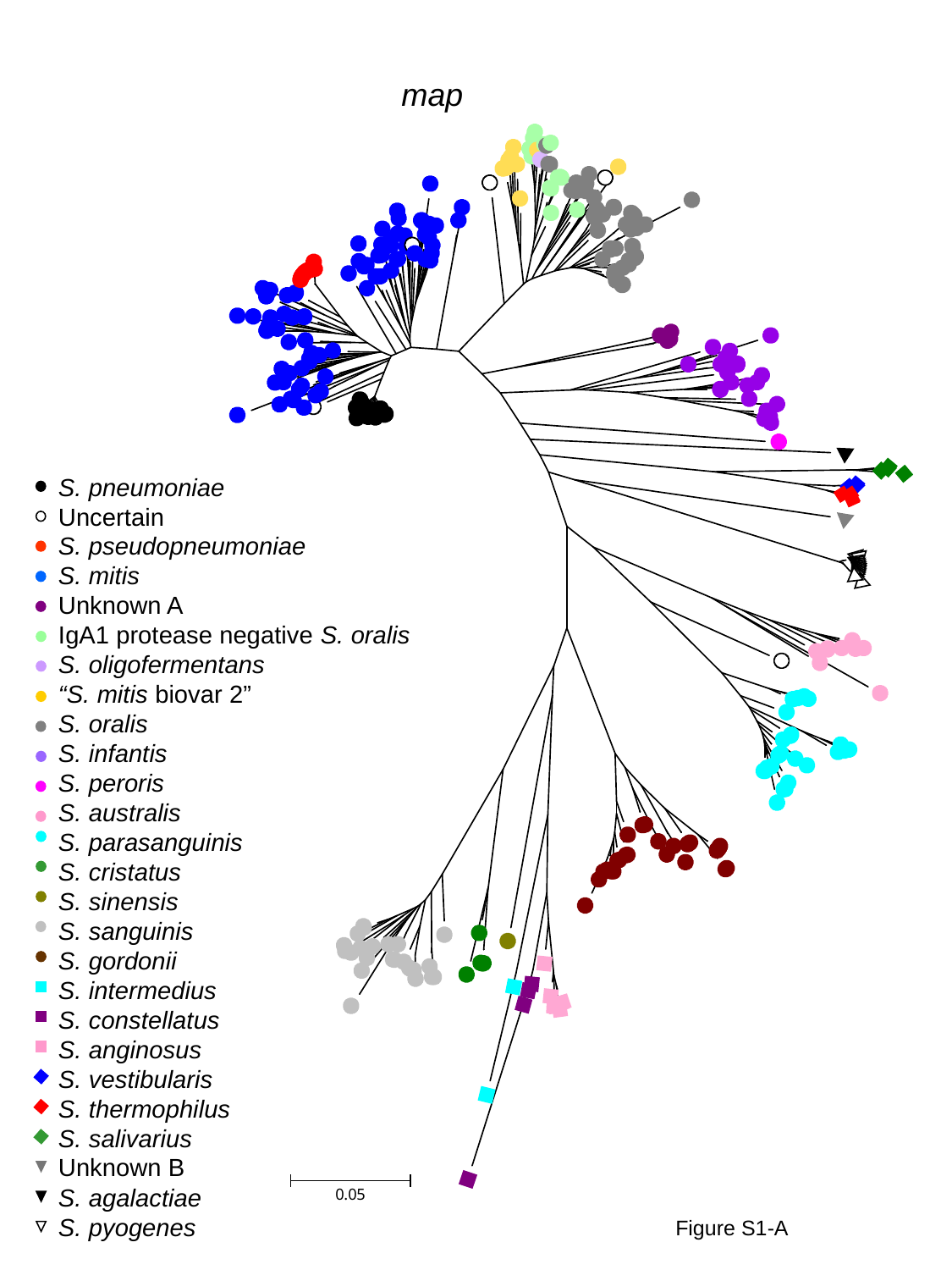

map
S. pneumoniae
Uncertain
S. pseudopneumoniae
S. mitis
Unknown A
IgA1 protease negative S. oralis
S. oligofermentans
“S. mitis biovar 2”
S. oralis
S. infantis
S. peroris
S. australis
S. parasanguinis
S. cristatus
S. sinensis
S. sanguinis
S. gordonii
S. intermedius
S. constellatus
S. anginosus
S. vestibularis
S. thermophilus
S. salivarius
Unknown B
S. agalactiae
S. pyogenes
Figure S1-A

## Slide 2
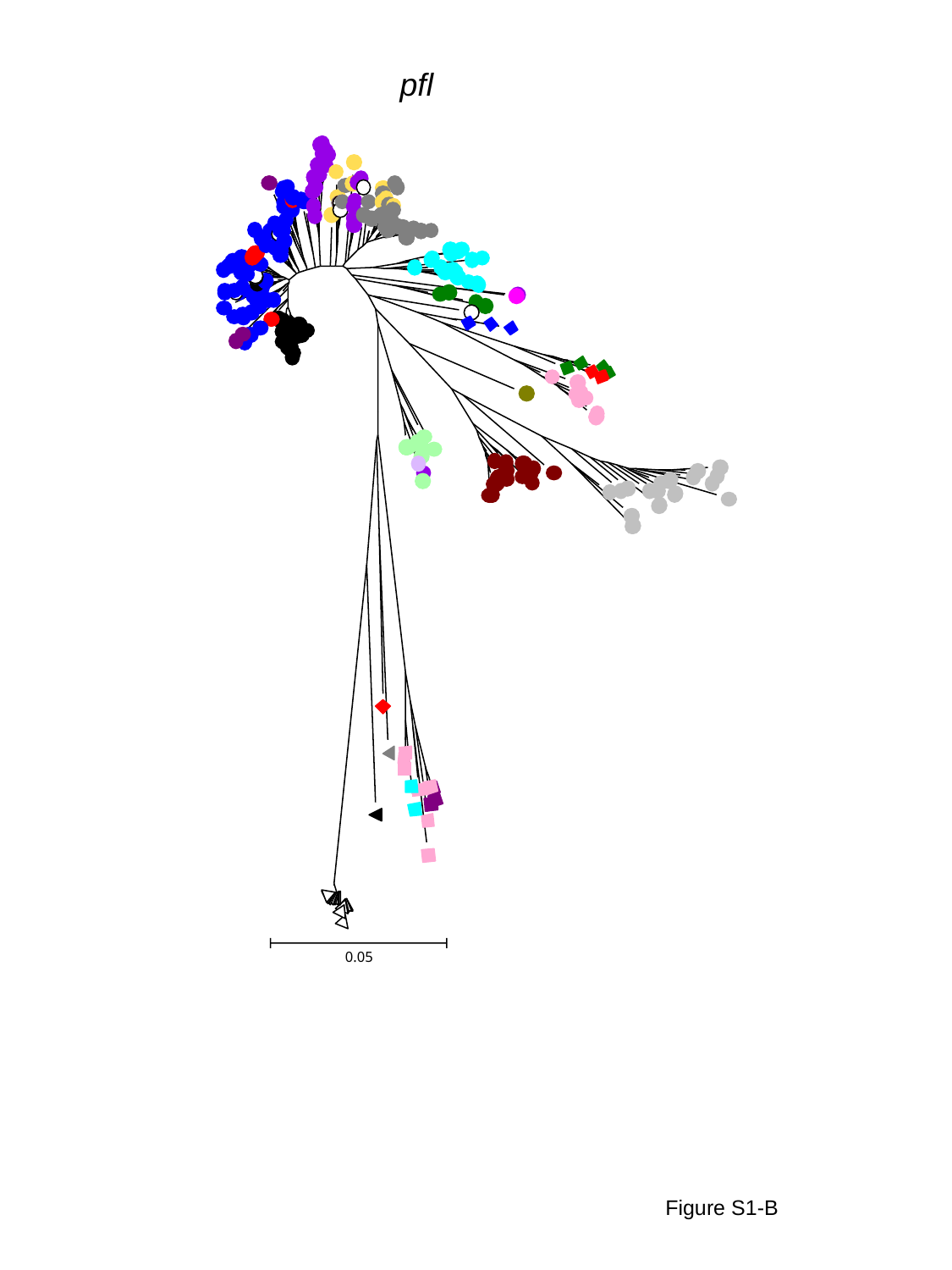

pfl
0.05
Figure S1-B

## Slide 3
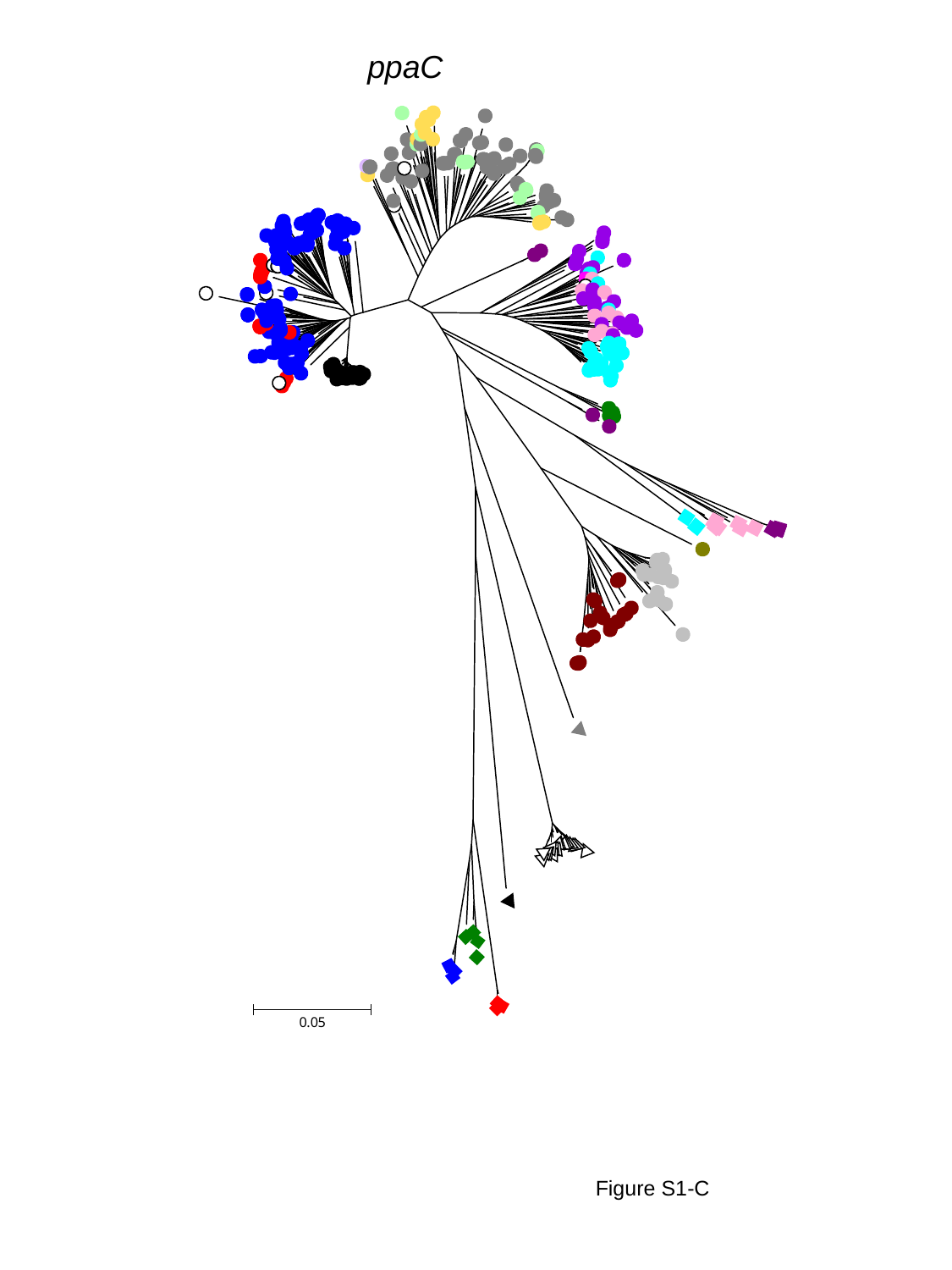

ppaC
Figure S1-C

## Slide 4
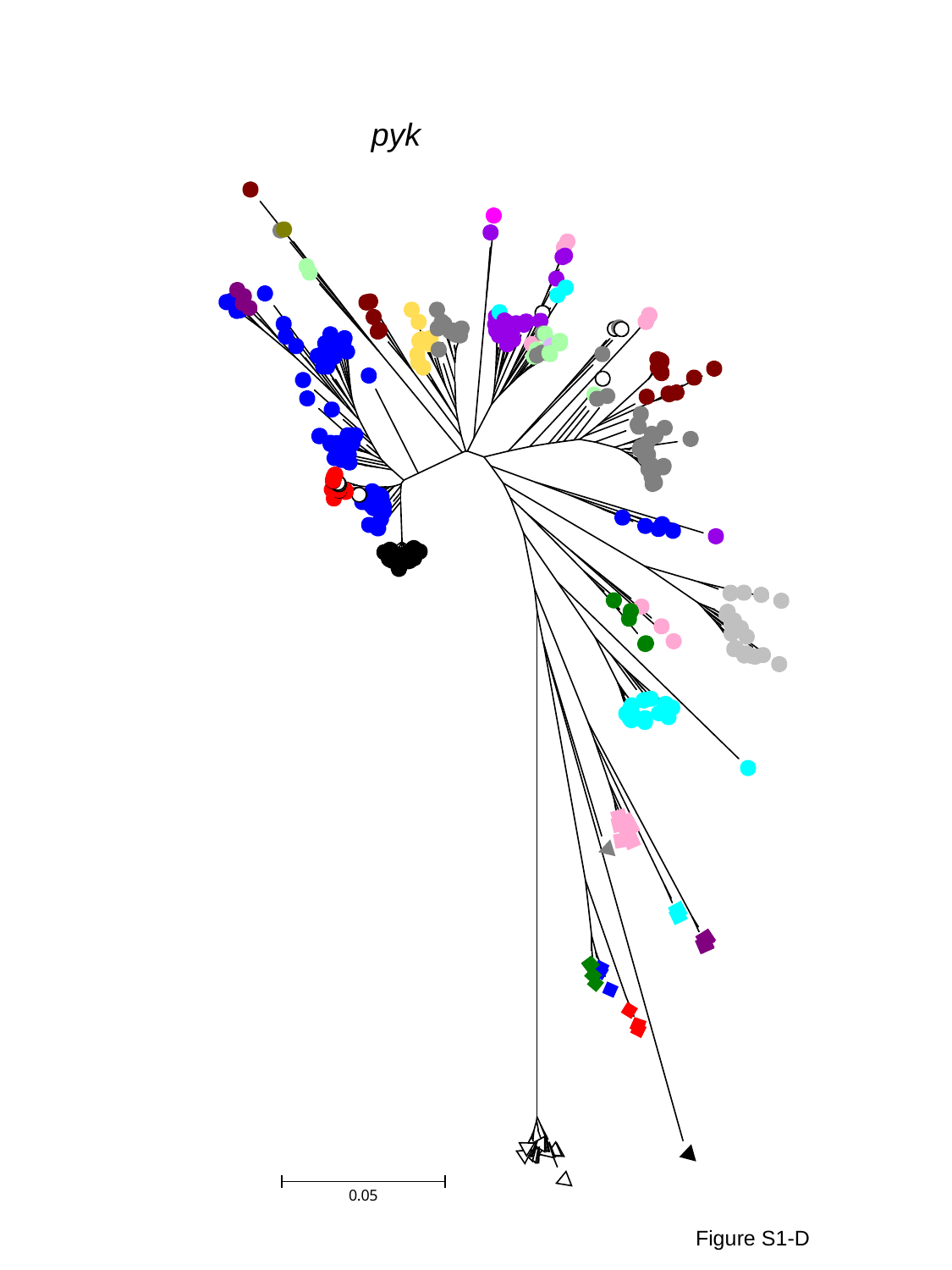

pyk
Figure S1-D

## Slide 5
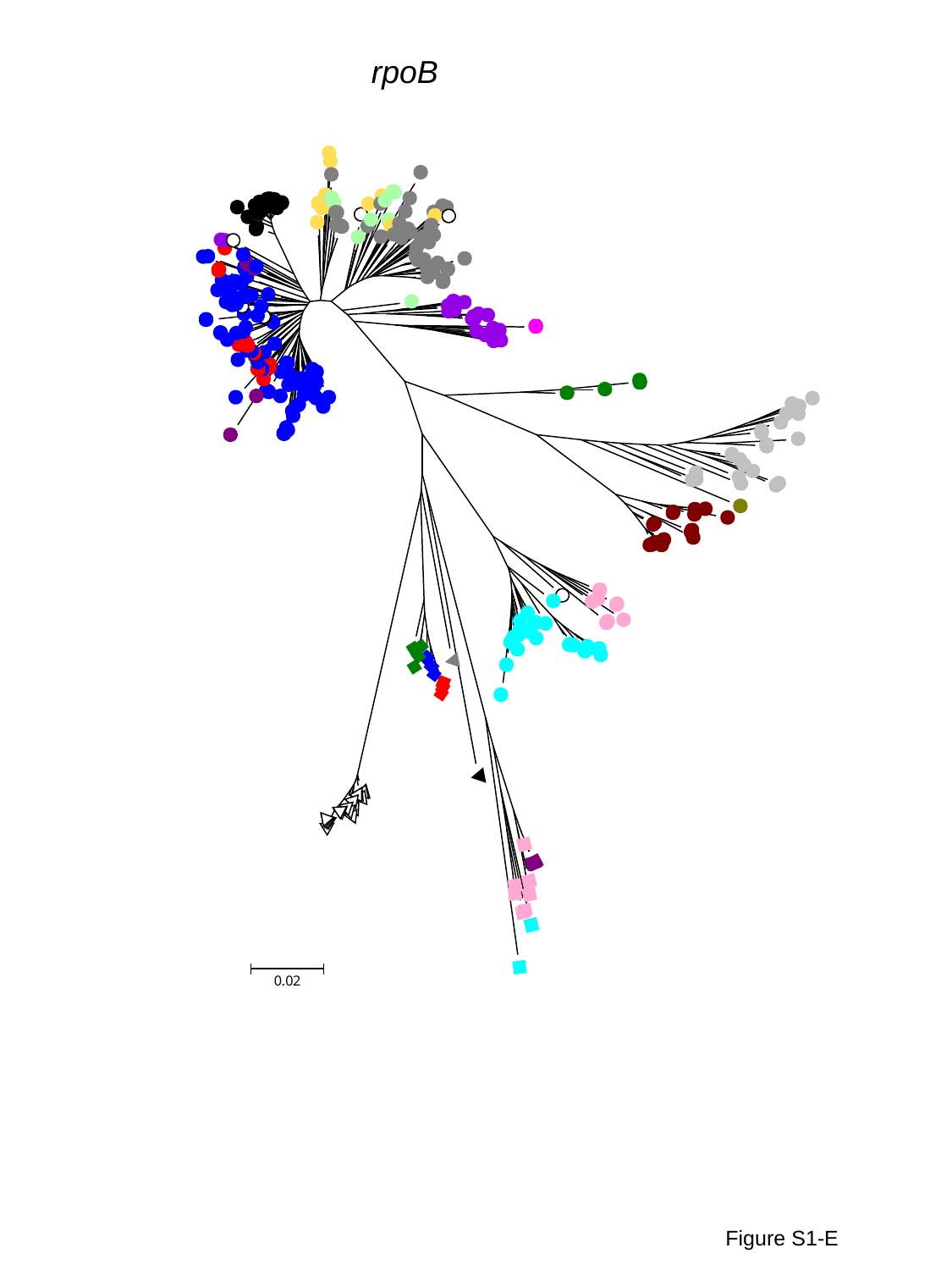

rpoB
Figure S1-E

## Slide 6
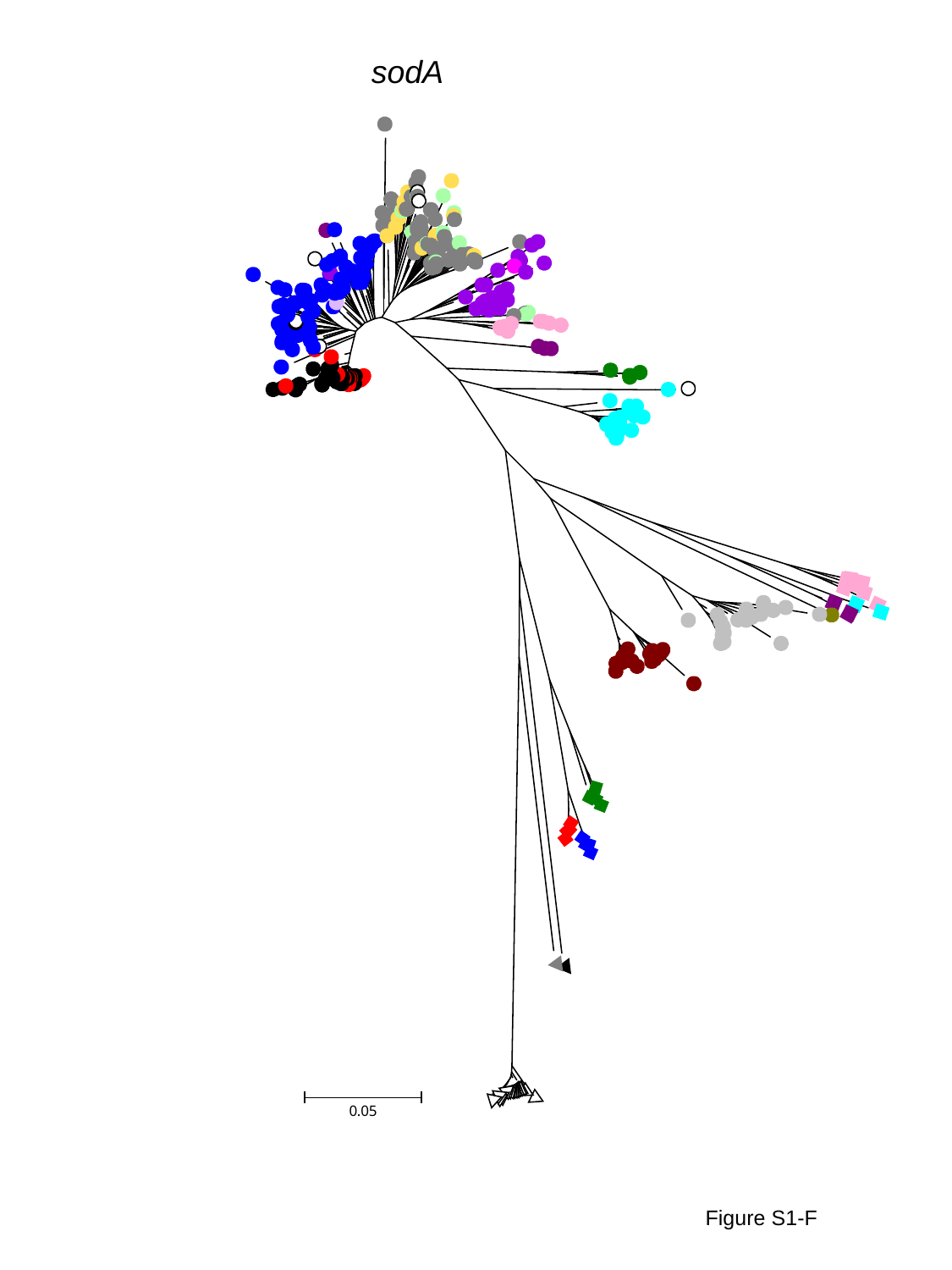

sodA
Figure S1-F

## Slide 7
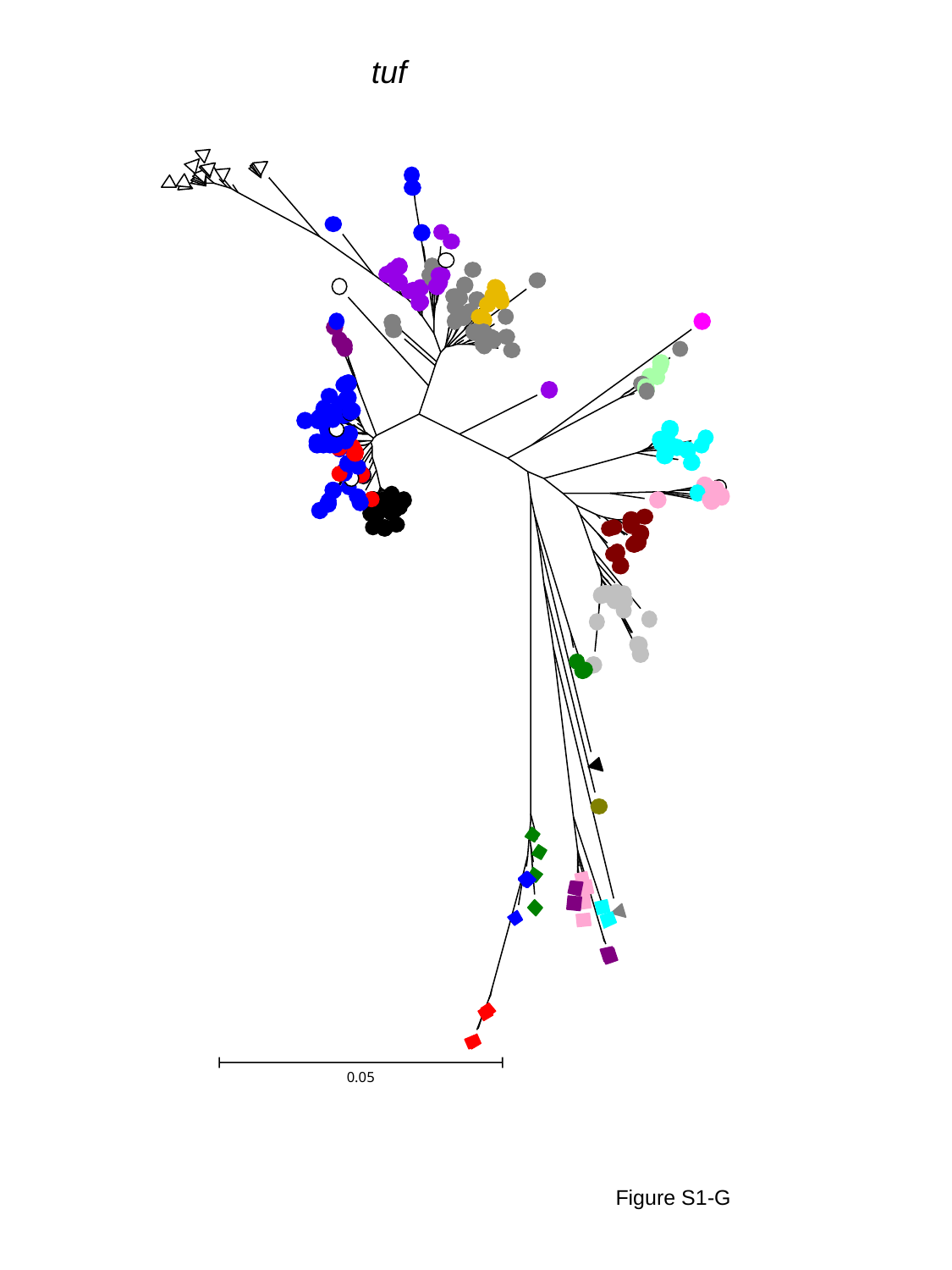

tuf
0.05
Figure S1-G
